# Supplementary material for: Performance, complexity and dynamics of force maintenance and modulation in young and older adults
Source: PLoS One. 2019 Dec 10;14(12):e0225925. doi: 10.1371/journal.pone.0225925 (PMC6903729; doi:10.1371/journal.pone.0225925)
Supplement: Attachment — Submitted filename: Response to reviewers.pdf [file pone.0225925.s001.pdf]

Rebuttal letter

We thank the editor and the three reviewers for their comments on our manuscript.

Please find below our response to each point raised by the academic editor and reviewers. We hope that we satisfyingly addressed all of them, and that the manuscript will be now suited for publication.

Sincerely,
On behalf of all authors,
Rita Sleimen-Malkoun

Academic editor:

No comments provided.

Reviewer #1:

The topic is interesting. However, there are some minor concerns might need to be clarified.

1. In page 4, the authors mentioned "we aimed for a broad characterization of force control under different task conditions in young and older adults. To do so we combined conventional statistical measures of performance (mean force, variance, coefficient of variation and root mean squared error) with measures of force signals' complexity (MSE) and dynamics (stochastic and deterministic components). .....", please describe the purpose more clearly.

To address the reviewer's concern, the paragraph was reformulated as follows:

"We aimed at characterizing force control capabilities and properties in young and older adults when accomplishing isometric force control tasks with different requirements. Therefore, we analyzed performance in terms of precision (mean force, variance, coefficient of variation and root mean squared error), as well as the complexity (MSE) and the dynamics (stochastic and deterministic components) of the produced behavior. In that regard, while the precision variables are of clinical relevance, and allow for comparison with other force production studies, they contain little to no information as to the underlying force control system's functional organization. In contrast, the complexity measure provides informs about the time scales involved in the processes underlying force production, whereas the analysis of the dynamics separates the deterministic, and thus lawful, functional component of the performance dynamics from stochastic fluctuations. These analyses are thus complementary in characterizing force production."

2. In page 5, the subjects divided into two group, which are young participants (YN: 18; mean age ± SD = 24.9 ± 3.56 years; 6 females), and elderly participants (EL: 12; mean age ± SD= 76.69 ±6.41 years; 8 females). Do the authors consider the gender effect?

Gender effects have not been directly considered in the present study. However, by normalizing the required force to the individual MVC, we avoided one known gender bias, which is grip force difference between men and women (Haward and Griffin (2002). Although grip force differences do exist, gender effects on finger coordination and relative force production were not previously reported (see for example Li, Latash, Zatsiorsky, 1998; Haward and Griffin, 2002; Latash, Danion, Zatsiorsky, 2002; Shinohara, Li, Kang, Zatsiorsky, Latash, 2003). Accordingly, we have no reasons to think that gender might have introduced a bias in our study.

3. Page 5, line 140, the term "LabView" should be "LabVIEW".

We have corrected LabView to LabVIEW.

4. How many trails for a subject and if they can familiar the trail before test recording?

As mentioned in the Task and Procedure section, each participant performed 6 experimental trials for each of the 6 task conditions, adding up to a total of 36 trials. These trials were preceded by 1 familiarization trial in each force production condition, totaling 6 familiarization trials. To be noted is that all participants were informed that they could run an additional familiarization trial if they did not feel comfortable with the task or the set-up. We have added the total number of trials for the familiarization and the experiment in the revised manuscript.

5. The "Results" section include the results of MVC, General characteristics of force performance, Multi Scale Entropy and Drift and diffusion coefficients. If the results can be present in tables, it would be better to realize the results.

We originally did not include a table to avoid redundancy with the text. We have included one now hoping it will aid depicting the results.

Table 1. Repeated measures ANOVA table of the significant results in the constant and sinusoidal force tracking tasks. The variables that were analyzed were the mean applied force, standard deviation (SD), coefficient of variation (CV), Root-Mean-Square-Error (RMSE), multi-scale entropy (MSE), drift and diffusion coefficients.

		**Constant force**			
Variable		*F*	*df*	*p*	η_{π}^2
**Mean force**	*Force*	227308	1,28	<.001	1
	Force×Age	8.85	1,28	<.01	0.24
**SD**	*Force*	156.98	1,28	<.001	0.85
	Age	6.26	1,28	.018	0.18
**CV**	*Force*	11.76	1,28	.002	0.3
	Age	5.67	1,28	.02	0.17
**RMSE**	*Force*	72.79	1,28	<.001	0.72
	Age	9.99	1,28	.004	0.26
**MSE**	*Age*	10.38	1,28	.003	0.27
**Drift coefficient**	*Bins*	246.94	1,30	<.001	0.9
	Force×Bins	89.6	1,39	<.001	0.76
**Diffusion coefficient**	*Force*	59	1,28	<.001	0.68
	Bins	40.64	1,34	<.001	0.59
	Force×Bins	27.67	1,40	<.001	0.5
		**Sinusoidal force**			
		F	*df*	*p*	η_{π}^2
**Mean force**	*Force*	35544	1,28	<.001	0.99
	Frequency	6.26	1,28	<.001	0.41
	Force×Age	27.46	1,28	<.001	0.5
	Age×Frequency	15.81	1,28	<.001	0.36
	Frequency×Force	7.47	1,28	.01	0.21
**Phase locking**	*Age*	4.54	1,28	.04	0.14
**Circular variance**	*Age*	71.8	1,28	<.001	0.72
	Force	24.61	1,28	<.001	0.47
	Frequency	47.72	1,28	<.001	0.63
	Age×Force	11.91	1,28	.002	0.3
	Force×Frequency	37.7	1,28	<.001	0.57
	Age×Force×Frequency	1.84	1,28	<.001	0.38
**RMSE**	*Age*	118.67	1,28	<.001	0.81
	Force	5.87	1,28	.02	0.17
	Frequency	21.21	1,28	<.001	0.43
	Force×Frequency	10.96	1,28	.003	0.28
	Age×Force×Frequency	8.11	1,28	.008	0.23
**MSE**	*Age*	11.66	1,28	.002	0.29
	Force	45.53	1,28	<.001	0.61
	Frequency	230.31	1,28	<.001	0.89
	Age×Force	8.72	1,28	.006	0.24
	Age×Frequency	5.96	1,28	.02	0.18
**Drift coefficient**	*Force*	6.57	1,28	.02	0.19
	Frequency	711.82	1,28	<.001	0.96
	Bins	215.9	4,101	<.001	0.89
	Age×Frequency	7.45	1,28	.01	0.21
	Age×Bins	9.8	3,101	<.001	0.26
	Force×Bins	5.19	5, 128	<.001	0.16
	Age×Force×Bins	4.74	5,128	.001	0.13
	Frequency×Bins	24.38	4,121	<.001	0.47
	Age×Frequency×Bins	4.08	4,121	.003	0.13
**Diffusion coefficient**	*Age*	18.68	1,28	<.001	0.4
	Force	15.32	1,28	.001	0.35
	Frequency	950.89	1,28	<.001	0.97
	Bins	232.63	3,103	<.001	0.89
	Age×Bins	17.07	3,103	<.001	0.38
	Force×Frequency	4.29	1,28	.048	0.13
	Force×Bins	8.53	5,143	<.001	0.23
	Age×Force×Bins	2.89	5,143	.02	0.094
	Frequency×Bins	26.33	4,115	<.001	0.49
	Age×Frequeny×Bins	4.13	4,115	.003	0.13
	Force×Frequency×Bins	2.87	4,111	.03	0.09

6. In page 12, the description "Fig 2. General characteristics of force production. Mean force in the constant task (a) and the modulation task (b). Standard deviation (c) and coefficient of variation (e) for the constant task. Mean relative phase (d) and uniformity (f) for the modulation task. Black bars represent the results of the young group, and white bars those of the elderly. Error bars represent the standard deviation." Maybe follow the order (a)….(b)….(c)…(d)….(e)….(f)…would be better.

We have adopted the reviewer's proposal and have re-written the figure legend as reported bellow. We also made sure that all the other figures' legends are coherent with this presentation.

Modified legend: "Fig 2. General characteristics of force production. (A) Mean force in the constant task and (B) the modulation task. (C) Standard deviation for the constant task. (D) Mean relative phase for the modulation task. (E) Coefficient of variation for the constant task. (F) Uniformity for the modulation task. Black bars represent the results of the young group, and white bars those of the elderly. Error bars represent the standard deviation."

7. In page 13, the description "Fig 3. Root mean square error (RMSE) of produced force. young (white) and elderly (black) participants in the constant"? It is different from that show in Fig. 3 young (black) and elderly (white).

We thank the reviewer for pointing out this inconsistency. It is now corrected.

8. In page 14, the description "Fig 4. Multiscale entropy of produced force. Mean sample entropy for constant (a) and modulation (b) tasks, for young (black) and elderly participants (black)" It is different from that show in Fig. 4 young (black) and elderly (white).

We thank the reviewer for pointing out this inconsistency. It is now corrected.

9. If the printing version is in black-and-white, the color figures should be revised.

We assure the reviewer that the manuscript will be printed in color.

10. Maybe the author can describe the application of their finding in the manuscript.

We have mentioned a potential application of our findings in gerontechnologies (lines 444-6 in the discussion of the submitted manuscript), but we acknowledge that this aspect was not sufficiently developed. Following the reviewer's recommendation, we added a paragraph in the conclusion section about possible applications:

"The better understanding of age-related force control deficits and their manifestation is of great relevance for healthcare interventions in the elderly, as well as the silver economy sector developing different gadgets that must meet the needs and the capabilities of the seniors. It is a well-established practice in geriatrics to test maximal grip force, which here we show that it does not account by itself for age-related declines in force control. With the development of telemedicine and actimetry directed to monitoring patients over longer periods of time with the use of wearable medical devices, the used paradigm and analysis methods could be integrated in a user-friendly interface to offer a more comprehensive assessment tool for clinicians. It can also be implemented with a gamification effort to train older adults and stimulate their force control capacities."

<u>Reviewer #2:</u>

1. Aging increased the stochasticity (noise-driven fluctuations) of force fluctuations in the cyclic force modulation, which could be related to the increased complexity found in the elderly for this same task. This findings of this study show their complementarity in revealing distinct aspects of sensorimotor adaptation to task constraints and age-related declines. Further research is still needed to identify the physiological underpinnings. The authors may make a paragraph to direct future works.

This is indeed a very important point that we attempted to address in the Conclusion sub-section (lines 626-29 in the submitted manuscript) by offering three directions for future studies: (i) examining a larger cohort of advanced age participants, (ii) making the link with physical and cognitive functioning, and (iii) adding electrophysiological recordings. However, it is more difficult to integrate in the abstract due to word count limitations (as we understand is suggested by the reviewer who copy-pasted a paragraph from the abstract).

2. The authors used two tasks requiring either force maintenance (straight line target force) or force modulation (sine-wave target force) around different force levels and at different modulation frequencies.

It is not easy to know the procedure of manipulation activities. The authors may consider to make a photo or sketch to illustrate the procedure of two manipulation activities. Figure 1 is not clear to know the detail.

We adapted Figure 1 and made the caption more explicit. We hope that the procedure would be clearer now.

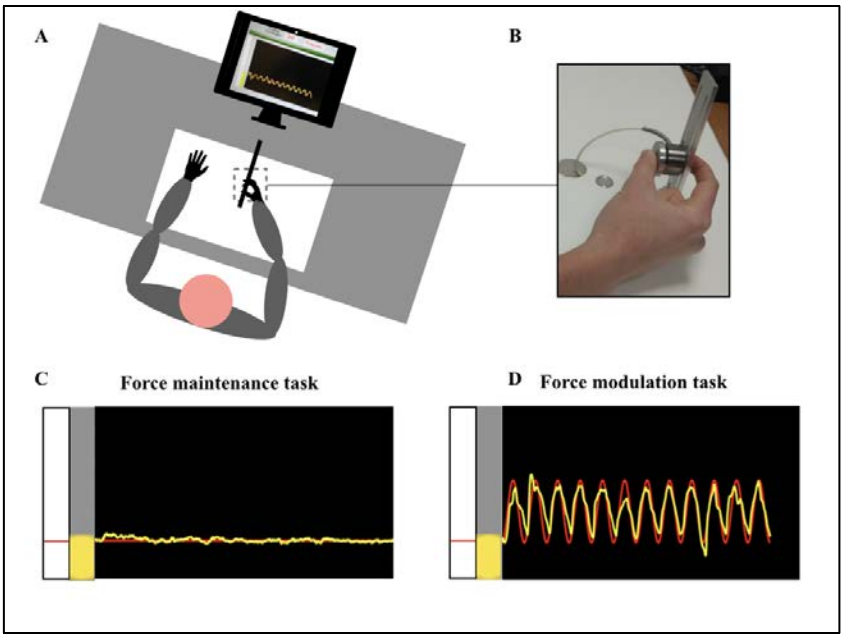

Fig 1. Experimental set-up. (A) Illustration of the experimental set-up showing the position of the participant facing the computer screen where the visual feedback is given, and holding the height-adjustable force transducer. (B) Close-up on a young participant holding the force transducer in a pinch-grip with their dominant hand. (C) Screenshot of the visual feedback during an exemplary constant force trial performance. (D) Screenshot during an exemplary sinusoidal force trial performance. The participants were required to squeeze the transducer (B) in order to move-up the on-screen yellow bar over the grey background, and match the red target line that appeared in the white space. Then, the red line would draw from left to right either as (C) a straight line or (D) a sine wave that the participant should keep matching with the yellow curve driven by their produced force.

3. A height-adjustable force transducer was affixed to the experimental table. The participant could comfortably grasp it while being seated with their arms resting on the table. The authors may make a photo to illustrate how the force transducer affixed to the table, and how the subjects comfortably grasp it. Figure 1 is not clear to know the detail.

As shown above, Figure 1 was modified to include a panel with a close-up on the force transducer.

4. The authors may list the analysis formula (i.e., root mean squared error, multiscale entropy) of this study. It would be easy to know how to calculate these parameters.

We have added the requested equations to the manuscript.

(1) $RMSE = \sqrt{\frac{1}{N-1}\sum_{i=1}^{N}(x_i - t)^2}$; where *N* is the number of data points in the time series, t is the force target, and x_i is the force sample.

(2) $CMSE = \frac{1}{\tau}\sum_{k=1}^{\tau} SampEn(y_k^{\tau}, m, r)$; where *k* is number of coarse-grained time-series for a scale factor of τ.

Reviewer #3:

The current findings set up a foundation for further study about motor control ability in people of different ages. However, the limitations of this study are not described so that readers might overinterpret the results.

We did mention three key limitations that need be addressed in future studies as we see them (cf. end of the conclusion section, lines 626-29 in the submitted manuscript). These three limitations are: (i) examining a larger cohort of advanced age participants (due to the great inter-individual variability within this group), (ii) making the link with physical and cognitive functioning, and (iii) adding electrophysiological recordings. Nevertheless, following the reviewer's comment, we reformulated the corresponding paragraph in the conclusion section to better emphasize this point:

"Our study presents some limitations that need to be addressed in future work. Examining for instance a larger cohort of advanced age participants would help in clarifying the effects that did not reach statistical significance, most likely due to the great inter-individual variability of the older group. Additionally, including participants with different levels of physical and cognitive fitness, as well as adding electrophysiological recordings, could help unravel eventual compensation strategies, as well as the moderators of a higher behavioral functioning."

In my opinion, the clinical application should be clarified to increase the value of the results.

We do believe that understanding age-related force control deficits and their manifestation is certainly relevant for a better health care in elderly. As explained in response to reviewer #1's last comment, we have now included a paragraph in the conclusion section wherein we mention the potential application of our findings.

MAJOR COMMENT

There is no need to put any term like "see", "as in" etc. before the citation number. Therefore, please delete all of them.

As requested, we have deleted "see" and "as in", except for: "see for an overview" and "for an illustration see".

Introduction. In general, the introduction section is very comprehensive. It seems like authors focus a lot on introducing the application of entropy-based metric and dynamics on physiological signals, indicating the complexity and dynamic aspects of signals. I suggest that the authors should emphasize more on the importance of applying these analysis methods to the medical signals, and the purpose of this study would be more convincing. Moreover, the aim of this study does not have to be conveyed in the first paragraph. The first paragraph just needs to reveal the signal complexity and dynamics are essential in evaluating human performance.

We are glad to know that we succeeded in positioning our work in a comprehensive manner despite the large number of concepts and methods we had to span. We chose conveying the aim of the study as early as the first paragraph to facilitate the understanding of the organization and the progression of the introduction.

We thank the reviewer for pointing-out the lack of mention of the clinical domain. Following their proposal, we now make reference in the introduction to some of the studies applying MSE in bipolar disorders (Nardelli et al. 2017, DOI: 10.1038/s41598-017-18036-z), diabetes (Costa et al., 2014, doi: 10.1063/1.4894537), and chronic obstructive pulmonary disease (Jin et al., 2017, doi: 10.2147/COPD.S140636. eCollection 2017). We also emphasize this aspect in the revised Conclusion section as part of the application perspectives of this study (see the response to reviewer #1, comment #10).

Results. After reading all statistic results, I am not certain how the authors performed ANOVA. Basically, the main result of ANOVA, which contains 2 or more factors, is if there is a significant interaction between factors first. Later the main effect of each factor could be examined. Last the post-hoc test would be carried out to make multiple comparisons. Take the result of constant force as an example. However, the authors reported there is a significant difference in the mean applied force between force levels first. Subsequently, the ANOVA shows an interaction between force level and age. Furthermore, the figure 2a seems not to have an interaction between age and force level. I am confused about what statistic analysis the authors used to derive the first and second result. Each variable which is reported later in this manuscript also confused me a lot.

The analysis of variance was performed in SPSS by specifying the factors described in the statistical analyses sub-section, and then the obtained results were reported following the APA style format with main effects reported first, followed by the interaction(s), and last post-hoc test results if needed. We however took the liberty of reporting only significant effects for sake of brevity. This might have been a confusing factor, although we had specified it in the statistical analyses sub-section. Beyond this, we fail see any other unconventionality in our

ANOVA. Since this point was not raised by the two other reviewers, we refrained from re-writing the whole results section, which would bring a substantial change in the presentation of the findings.

Discussions. The discussion section is well written. I consider it might be better that the first two paragraphs of conclusion are summarized in the discussion section. Also, please discuss the limitations of this study.

We understand the point of the reviewer regarding the first two paragraphs of the conclusion. However, since the discussion is organized in sub-sections, we deem it more appropriate to keep the summary of the discussed results in the conclusion.

With regard to the limitations of the study, as aforementioned in our response to the first comment of reviewer #3, it is now covered by the last paragraph of the Conclusion.

MINOR COMMENT

Introduction. Line 100: Please add the full name of "NMSS" when first using.

We have changed NMSS into neuro-musculo-skeletal system, as used in the rest of the manuscript.

Methods. Line 131-134: How did you define young and elderly participants? And please report body weight and height of all participants.

The participants groups were defined based on their chronological age, with the elderly being older than 65 years (ranging from 66 to 85 years), and the young adults being older than 18 years and younger than 35 years (ranging from 19 to 34). We have now added this information the "Participants" sub-section.

With respect to body weight and height, this information was only collected in the initial enrolment phase to insure that none of our participants was over or under weight. To our knowledge, it is not a common practice to report weight/height information in force control studies, especially so when force requirements are normalized to the individual maximum voluntary contraction and the setup is height-adjustable. We therefore fail to grasp the significance of the reviewer's request.

Line 244: Are t-tests used as post hoc tests? Please specify what test you used for post hoc tests.

We have indeed used corrected t-tests as post hoc tests, and have specified this in the submitted manuscript in the statistical analyses section (line 244).

Results. Some p values here are reported to be 0.000. The p value is impossible to be 0.000, so please revise all of them into "p<0.001".

We thank the reviewer for pointing out this inconsistency and have corrected the p-values.

Line 258: "p=.0018": Based on other p values reported, if the authors want to present the value with three decimal places, please be consistent. "0.0018" should be round to three decimal places

We have corrected the p-value to $p = .002$.

Line 388, Line 403 and Line 407: I guess "bins [3, 8]" indicates bin is 3 and 8. But the square brackets confuse readers because brackets are also used for citation. Please use other symbols or expressions.

We thank the reviewer for pointing this out and have removed the square brackets.

Figure 2. Please add the full name of MVC in legend.

Figure 3. Please add the full name of MVC in legend.

Figure 4. Please add the full name of MVC and CMSE in legend.

Figure 5. Please add the full name of MVC in legend.

Figure 6. Please add the full name of MVC in legend.

As requested we have added the full name of MVC in all the legends. CMSE was already spelled out in Fig.4.
