# Supplementary material for: Performance, complexity and dynamics of force maintenance and modulation in young and older adults
Source: PLoS One. 2019 Dec 10;14(12):e0225925. doi: 10.1371/journal.pone.0225925 (PMC6903729; doi:10.1371/journal.pone.0225925)
Supplement: Attachment — Submitted filename: Response to reviewers.docx [file pone.0225925.s002.docx]

Please find below our response to the suggestion made by reviewer #2 and the last remaining point raised by the reviewer #3.

**Reviewer #2:**

***This is a simple task for young and older adults. Authors may consider making an interesting story or purpose to promote this task for reader reading attractively.***

Following the reviewer’s suggestion, we now put more forward the functional and clinical relevance of the task, by adding the following to what was already mentioned in the conclusion section.

- End of the abstract: “*Although further research is still needed to identify the physiological underpinnings, the used task and methodology is shown to have both fundamental and clinical applications*.”
- First paragraph of the introduction: “*In the present work, we adopted a broad perspective to characterize force control in young and older adults in a functionally relevant task that can be quantitatively manipulated to scale the imposed attentional and sensorimotor requirements*.”
- First paragraph of the discussion: “*The used force control task involves multiple control processes (e.g., force production, visuomotor integration, and attention) that are implicated in daily living activities, and presents also the advantage of being easy to implement and undergo for both research and clinical purposes*.”

**Reviewer #3:**

***Thanks for authors' response. I still have one more question from your revision.***

***I am not sure your definition of "the corrected t-test" used in this study as the post hoc test. I guess you probably directly used the bonferroni test in SPSS to perform multiple comparisons so that you don't need to report the adjusted p value. But if using t-tests with an adjusted significant level (we call it as bonferroni correction), you must report adjusted p value. Either way is correct. Please specify.***

The reviewer’s guess is correct. We did use the Bonferroni test in SPSS. This is now clearly specified in the methods section.
